# Climate change is associated with increased allocation to potential outcrossing in a common mixed mating species

**DOI:** 10.1002/ajb2.16021

**Published:** 2022-06-28

**Authors:** Matthew W. Austin, Piper O. Cole, Kenneth M. Olsen, Adam B. Smith

**Affiliations:** ^1^ Living Earth Collaborative Washington University in St. Louis St. Louis MO USA; ^2^ Division of Natural Sciences New College of Florida Sarasota FL USA; ^3^ Department of Biology Washington University in St. Louis St. Louis MO USA; ^4^ Center for Conservation and Sustainable Development Missouri Botanical Garden St. Louis MO USA

**Keywords:** dimorphic cleistogamy, floral dimorphism, global change, North America, phenological shift, PRISM, *Viola*, Violaceae, violets, water availability

## Abstract

**Premise:**

Although the balance between cross‐ and self‐fertilization is driven by the environment, no long‐term study has documented whether anthropogenic climate change is affecting reproductive strategy allocation in species with mixed mating systems. Here, we test whether the common blue violet (*Viola sororia*; Violaceae) has altered relative allocation to the production of potentially outcrossing flowers as the climate has changed throughout the 20th century.

**Methods:**

Using herbarium records spanning from 1875 to 2015 from the central United States, we quantified production of obligately selfing cleistogamous (CL) flowers and potentially outcrossing chasmogamous (CH) flowers by *V. sororia*, coupled these records with historic temperature and precipitation data, and tested whether changes to the proportion of CL flowers correlate with temporal climate trends.

**Results:**

We find that *V. sororia* progressively produced lower proportions of CL flowers across the past century and in environments with lower mean annual temperature and higher total annual precipitation. We also find that both CL and CH flower phenology has advanced across this time period.

**Conclusions:**

Our results suggest that *V. sororia* has responded to lower temperatures and greater water availability by shifting reproductive strategy allocation away from selfing and toward potential outcrossing. This provides the first long‐term study of how climate change may affect relative allocation to potential outcrossing in species with mixed mating systems. By revealing that CL flowering is associated with low water availability and high temperature, our results suggest the production of obligately selfing flowers is favored in water limited environments.

Climate change is affecting plant species in myriad ways, including effects on reproduction. As a changing climate alters regional weather patterns, altered growing seasons affect seedling recruitment (Mondoni et al., 2015; Hacket‐Pain and Bogdziewicz, 2021) and drive shifts in reproductive phenology (Menzel et al., 2006; Miller‐Rushing and Primack, 2008). Less known, however, is how climate change affects relative allocation to potential outcrossing vs. selfing in species with mixed mating systems, i.e., species where fertilization can occur via either outcrossing or selfing (Goodwillie et al., 2005). This is vital to understand because reduced outcrossing rates can lead to inbreeding depression and increased extinction risk in changing environments (Stebbins, 1957; Beattie, 1976; Solbrig, 1976). Experimental climate manipulations (Jones et al., 2013) and environmental associations across spatial gradients (Ansaldi et al., 2018) have suggested that reproductive strategy allocation may be labile under a temporally changing climate. Nonetheless, a lack of long‐term time series data across the 20th century has precluded documentation of whether climate change is associated with shifts in reproductive strategy allocation in species with mixed mating systems.

A classic theory for the maintenance of mixed mating systems posits that outcrossing offers the benefit of generating genetically diverse progeny, while selfing provides reproductive assurance in environments unsuitable for outcrossing (Darwin, 1877; Campbell et al., 1983; Oakley et al., 2007). Under this framework, outcrossing and selfing have optima under different environmental conditions; outcrossing is employed in favorable environments where resources are sufficient to support energetic investment in outcross reproduction, while selfing is used as an adaptive strategy in stressful environments with lower resource availability (Campbell et al., 1983; Lloyd, 1984; Berg and Redbo‐Torstensson, 1998; Oakley et al., 2007). Mounting evidence suggests that climate change has altered levels of climatic stress experienced by plants (e.g., Ma et al., 2019). For example, for some species, increased climatic stress has resulted in populations being pushed near their thermal limits (Pörtner and Farrell, 2008) or occurring in environments depleted of essential resources (McCluney et al., 2012). For others, decreased climatic stress has induced more favorable growing conditions (Mondoni et al., 2015) that can support greater investment in overall reproductive output (Anderegg et al., 2021). Accordingly, if climate change shifts environmental conditions from the optimum of one reproductive strategy to the other by altering levels of climatic stress, then species with mixed mating systems might respond by altering their relative allocation to potential outcrossing.

Dimorphic cleistogamy—a type of mixed mating that occurs in 287 species across 56 angiosperm families (Lord, 1981)—is an ideal system for testing the effect of climate change on reproductive strategy allocation. In species with dimorphic cleistogamy, a single individual can produce two types of morphologically distinct flowers: cleistogamous (CL) flowers that obligately self‐pollinate and chasmogamous (CH) flowers that potentially (i.e., facultatively) outcross. CL flowers remain in a bud‐like stage that is structurally reduced for self‐fertilization (e.g., apetalous, absent of nectar, with reduced stamens and pistils), making them energetically cheaper to produce than CH flowers (Berg and Redbo‐Torstensson, 1998). CH flowers, on the other hand, produce elaborate floral displays and nectar rewards to attract animals as pollen vectors (Berg and Redbo‐Torstensson, 1998).

Trade‐offs between CL and CH flower production have been well documented. A critical trade‐off between them is the risk of inbreeding depression from obligately selfing CL flowers relative to the higher energetic cost of producing potentially outcrossing CH flowers (Schemske, 1978; Schoen and Lloyd, 1984; Goodwillie et al., 2005; Culley and Klooster, 2007; Oakley et al., 2007). Compared to hermaphroditic species with monomorphic flowers, dimorphism between CL and CH flowers allows reproductive strategy allocation to be readily quantified as the proportion of CL flowers that a plant produces. A higher proportion of CL flowers indicates greater investment in selfing relative to potential outcrossing (Eckert et al., 2010; Campbell et al., 2016; Ansaldi et al., 2018). Fitting with the hypothesis that selfing evolved as an adaptive strategy in stressful environments (Campbell et al., 1983; Lloyd, 1984; Berg and Redbo‐Torstensson, 1998; Oakley et al., 2007), environmental correlates of CL and CH flower production have been found in numerous species (e.g., Brown, 1952; Antlfinger et al., 1985; Bell and Quinn, 1986; Culley, 2002; Cortés‐Palomec and Ballard, 2006; Culley and Klooster, 2007; Campbell et al., 2016; Miranda and Vieira, 2016; Ansaldi et al., 2018). For example, in *Viola* (Violaceae)—a genus in which cleistogamy commonly occurs (Culley and Klooster, 2007)—CH flowers are often produced as temperatures rise in the spring, with CL flower production continuing as canopy closure reduces light availability (Culley, 2002). Furthermore, while interspecific variability exists in species with dimorphic cleistogamy (e.g., Campbell et al., 2016; Seguí et al., 2021), obligately selfing CL flowers are often produced in environments with low water availability and high temperature, suggesting that drought conditions are climatically stressful for many dimorphic cleistogamic species (e.g., Brown, 1952; Jones et al., 2013; Miranda and Vieira, 2016; Ansaldi et al., 2018).

Here, we test whether climatic changes across the 20th century are associated with a shift in reproductive strategy allocation in a species with a mixed mating system. We accomplish this by studying the common blue violet (*Viola sororia*), a dimorphic cleistogamic perennial native to eastern North America, an area that has experienced marked effects of climate change (EPA, 2016; NOAA, 2021). To ascertain how reproductive strategy allocation has varied with temporal changes in the climate, we quantified CL and CH flower production from central United States herbarium records spanning 1875–2015 and paired these records with historic climate data. We asked two questions: (1) Is climate change associated with a shift in reproductive strategy allocation? (2) Is climate change associated with a shift in the time of year that CL and CH flowers occur? Interestingly, our results suggest that climate change has altered the environmental conditions that *V. sororia* experiences, which has increased relative allocation to the production of potentially outcrossing CH flowers. By leveraging herbarium records as time series data, this presents the first long‐term study of how climate change correlates with reproductive strategy allocation in species with mixed mating systems.

## MATERIALS AND METHODS

### Study system

**Figure 1:**
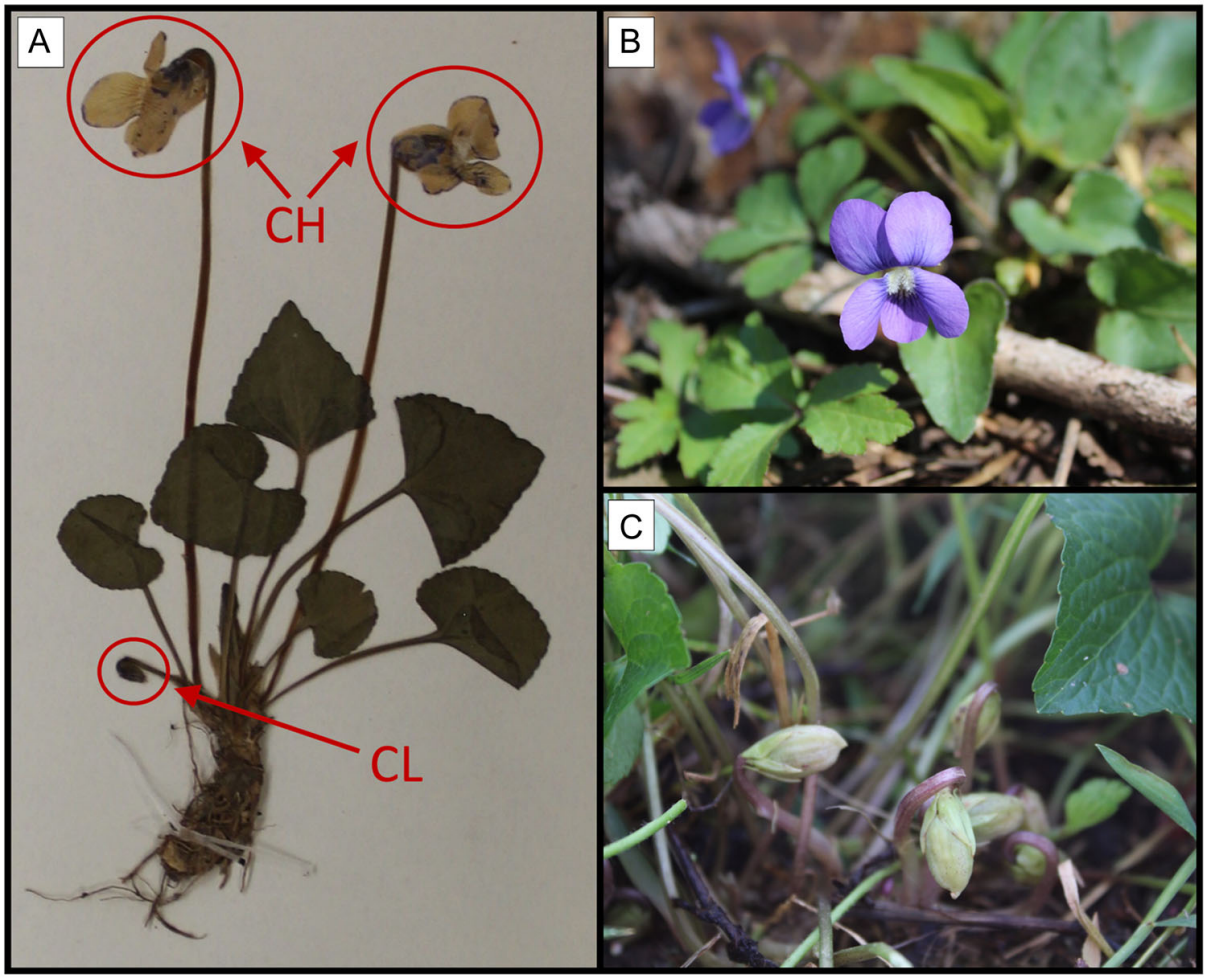
Dimorphic cleistogamy of *Viola sororia*; chasmogamous (CH) flowers may outcross, while cleistogamous (CL) flowers obligately self‐fertilize. (A) Herbarium specimen exhibiting CH and CL flowers. (B) Open CH flowers and (C) developing CL fruits of in situ *V. sororia* plants.

*Viola sororia* is a stemless herbaceous perennial violet native throughout eastern North America (Appendix S1), which is characterized by dimorphic cleistogamy and a predominantly subterranean rhizome yielding a rosette of leaves (Solbrig et al., 1980; Solbrig, 1981). Potentially outcrossing chasmogamous (CH) flowers produce zygomorphic, blue‐purple petals, while obligately selfing cleistogamous (CL) flowers are apetalous and lack color (Figure 1). In addition to the presence or absence of petals, a conspicuous distinguishing characteristic between the two flower types is peduncle length; CH flowers occur on long, erect peduncles that place flowers at or above leaf height, whereas CL flowers occur on short peduncles near the base of the rosette (Steyermark and Yatskievych, 1999; Culley and Klooster, 2007) (Figure 1). CH flowers are visited by a variety of pollinator taxa, with solitary bees often constituting the primary pollinator group (Beattie, 1974). Production of CH and CL flowers is largely sequential, with the main period of CH flowering occurring in the spring, and the main period of CL flowering in the summer and fall. However, similar to other violet species (Cortés‐Palomec and Ballard, 2006; Wang, 2013; Seguí et al., 2021), the transition between these two flower types is gradual, with overlapping production of CH and CL flowers occurring during this transition period. Occasional fall blooms of CH flowers can also occur (Austin, unpublished data), a phenomenon that occurs in other temperate spring ephemerals (e.g., Hopkins, 1937). All individuals are capable of producing both flower types (Solbrig, 1981).

**Figure 2:**
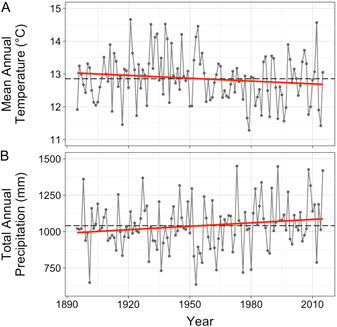
Temporal changes to Missouri's climate across the 20th century. Observations shown in gray are state‐wide averages of (A) mean annual temperature (in degrees Celsius, °C) and (B) total annual precipitation (in millimeters, mm) for 1000 coordinates randomly sampled across Missouri. Red lines give linear regression lines. Dashed black lines show the average climate values across this time period. Climate data derive from the Parameter‐elevation Regressions on Independent Slopes Model (PRISM) (Daly et al., 2002).

We focus the spatial extent of our study in Missouri, a state that occurs in the eastern central portion of the United States (Appendix S1) and exhibits a seasonal temperate climate (Appendix S2). Notably, across the past century, the eastern two‐thirds of the US has experienced higher annual precipitation (NOAA, 2021) and, although mean annual temperature has increased on average across the country, temperature has decreased in certain areas near the central United States (e.g., Banaszak et al., 2020; NOAA, 2021). In Missouri, when described by linear trends, total annual precipitation has increased by 7.9 mm (±4.2 SE) decade^−1^ and mean annual temperature has decreased by 0.029°C (±0.019 SE) decade^−1^ from 1895 to 2015 (Figure 2). However, linear trends belie the temporally heterogeneous change in climate across the state, which has oscillated between a warmer, drier period prior to the middle of the 20th century, followed by a more recent cooler, wetter period.

### Herbarium data

We collected data on *V. sororia* flowering phenology in Missouri from the herbarium of the Missouri Botanical Garden (MO). In addition to having experienced temporal climate changes across the 20th century, Missouri presents an ideal study area because of a predominance of *V. sororia* records at MO collected throughout the state. This is owing to a strong history of botanical collecting in Missouri since MO's founding in 1859.

To quantify dimorphic cleistogamy, we counted the total number of both CL and CH flowers per herbarium sheet and categorized each flower by phenophase (bud or fruit for CL flowers; and bud, open flower, or fruit for CH flowers). We used petal presence or absence and peduncle length to distinguish the two flower types; CL flowers lack petals and occur on short peduncles near the base of the rosette, whereas CH flowers and buds have petals and occur on long peduncles at or above leaf height. Two independent observers scored each herbarium record, and we discarded any records that were difficult to score or lacked agreement across observers. We calculated the proportion of CL flowers per herbarium sheet as the fraction of all flowers that were CL. Thus, the proportion of CL flowers ranged from 0 to 1, where 0 indicated that an herbarium sheet exhibited only CH flowers, and 1 indicated an herbarium sheet with only CL flowers. Accordingly, 1 minus the proportion of CL flowers gave the proportion of potentially outcrossing flowers on an herbarium sheet. Importantly, as CH flowers are facultatively—rather than obligately—outcrossing, CH flower production represents allocation to potential outcrossing, as opposed to realized outcrossing rates (e.g., see Schoen and Brown, 1991). We did not score phenology for specimens that lacked flowers of either type or were missing either locality data (i.e., state and county) or collection date (i.e., year, month, day) on the label. Because *V. sororia* flowers originate directly from the rhizome, we also did not score specimens that lacked an intact rhizome. In addition, because specimens contained on herbarium sheets may be portions of individual plants or multiple conspecific individuals mounted together, we consider each herbarium datum to be a snapshot of the population's phenology at a moment in time, without explicitly regarding individual‐level measurement.

### Climate data

To ascertain the historic climate for each of our herbarium records, we matched each record to temperature and precipitation data using the Parameter‐elevation Regressions on Independent Slopes Model (PRISM) at a 30 arcsecond resolution (Daly et al., 2002). PRISM is an interpolated weather product based on data across multiple monitoring networks for the coterminous United States from 1895 to the present (at the time of publishing this article), and is one of the most used interpolated weather data products for the United States (PRISM Climate Group, 2004). For each record, we used R (version 4.1.0) to randomly sample 1000 coordinates throughout its county of collection using the *dismo* (Hijmans et al., 2011) and *sp* (Pebesma and Bivand, 2005; Bivand et al., 2013) packages. We then used the *airUpThere* package (www.github.com/adamlilith/airUpThere) to match each of these 1000 coordinates to mean monthly temperature (in degrees Celcius) and total monthly precipitation (in millimeters) data for each month from July in the year prior to collection to June in the year of collection. Subsequently, we calculated the mean across these 1000 coordinates per variable and month. We then calculated mean annual temperature and total annual precipitation by respectively taking the mean of monthly mean temperatures and the sum of monthly total precipitation ranging from July in the year prior to collection to June in the year of collection. Accordingly, our climate data represent county‐wide measures of temperature and precipitation spanning a year from the summer prior to collection through spring in the year of collection, thus capturing weather in the spring reproductive season and the preceding winter and fall (Banaszak et al., 2020). We matched each specimen sheet to the climate trend in their county of collection.

### Statistical analyses

**Figure 3:**
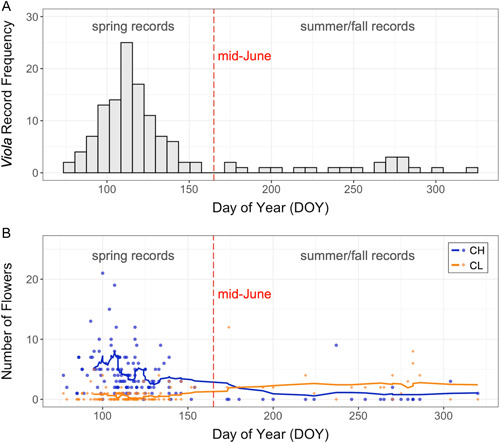
*Viola sororia* herbarium records. (A) The frequency of herbarium records across the growing season. (B) The occurrence of chasmogamous (CH) and cleistogamous (CL) flowers across the growing season. The dashed red line indicates mid‐June, which separates the spring reproductive season from the summer‐fall reproductive season. The solid blue and orange lines give the two‐week (14‐day) moving average for CH and CL flowers, respectively.

We restricted our analyses to records from late‐March through early‐June, because this corresponds to the time of year when *V. sororia* plants produce both CH and CL flowers as they transition from predominantly CH flowering to predominantly CL flowering. This is a conservative approach that prevents late season CL‐biased records from influencing our results and enables our findings to reflect proportional allocation to potential outcrossing during the initial reproductive season post winter dormancy. Furthermore, most herbarium records were collected prior to mid‐June (Figure 3A). To avoid pseudoreplication, we included no more than one herbarium record for each unique pairing of county, year, and day of year (DOY) in all models. When two or more herbarium records were collected in the same county and on the same date, we randomly selected one record that was retained for analysis. We used R (version 4.1.0) for all analyses.

#### Is climate change associated with a shift in reproductive strategy allocation?

To test whether climate change is associated with changes to the proportion of CL flowers, we ran two models: (1) a temporal model testing whether the proportion of CL flowers has changed across the past century, and (2) a climate model testing whether the proportion of CL flowers is associated with precipitation and temperature. For studies that leverage long‐term data to ascertain the effect of climate change on phenology via correlative approaches, it is standard practice to analyze the effect of time separately from the effect of climate, given autocorrelation between year and climate in the era of anthropogenic climate change (Jones and Daehler, 2018). We ran each of these models as a generalized linear model (GLM) with the proportion of CL flowers as the response variable and a quasibinomial family to accommodate a proportion as the response. In the temporal model, we included year of collection as the predictor variable. In the climate model, we included mean annual temperature, total annual precipitation, and the interactions of these as predictor variables. We centered and scaled all independent variables in the climate model.

#### Is climate change associated with a shift in the time of year that CL and CH flowers occur?

To test whether climate change is associated with changes to the time of year that CL and CH flowers occur, we similarly ran a temporal model and a climate model per flower type. Each temporal model is a GLM regressing flowering DOY against year of collection. One of these GLMs analyzes the effect of year on the timing of spring CL flowers and is restricted to specimens containing at least one CL flower bud. The other GLM analyzes the effect of year on the timing of spring‐blooming CH flowers and is restricted to specimens containing at least one open CH flower. For our climate models, we ran two GLMs that regressed flowering DOY against mean annual temperature, total annual precipitation, and the interactions of these. We performed a separate model for each flower type. For each climate model, we centered and scaled all independent variables. Note that all climate models are restricted to specimens collected in 1895 onward (Appendix S3), because 1895 is the earliest year for which PRISM data are available.

## RESULTS

### Herbarium records

We collected data on 131 *Viola sororia* records spanning 1875–2015, a period of 141 years. The majority of these records were collected in the spring, with 110 (~84%) collected between mid‐March (DOY 77) and early‐June (DOY 154) (Figure 3A); this is the timeframe to which we restricted our final analyses. Both obligately selfing CL and potentially outcrossing CH flowers occur across this entire spring period; our earliest and latest spring records each exhibited both CL and CH flowers (Figure 3B).

### Is climate change associated with a shift in reproductive strategy allocation?

**Figure 4:**
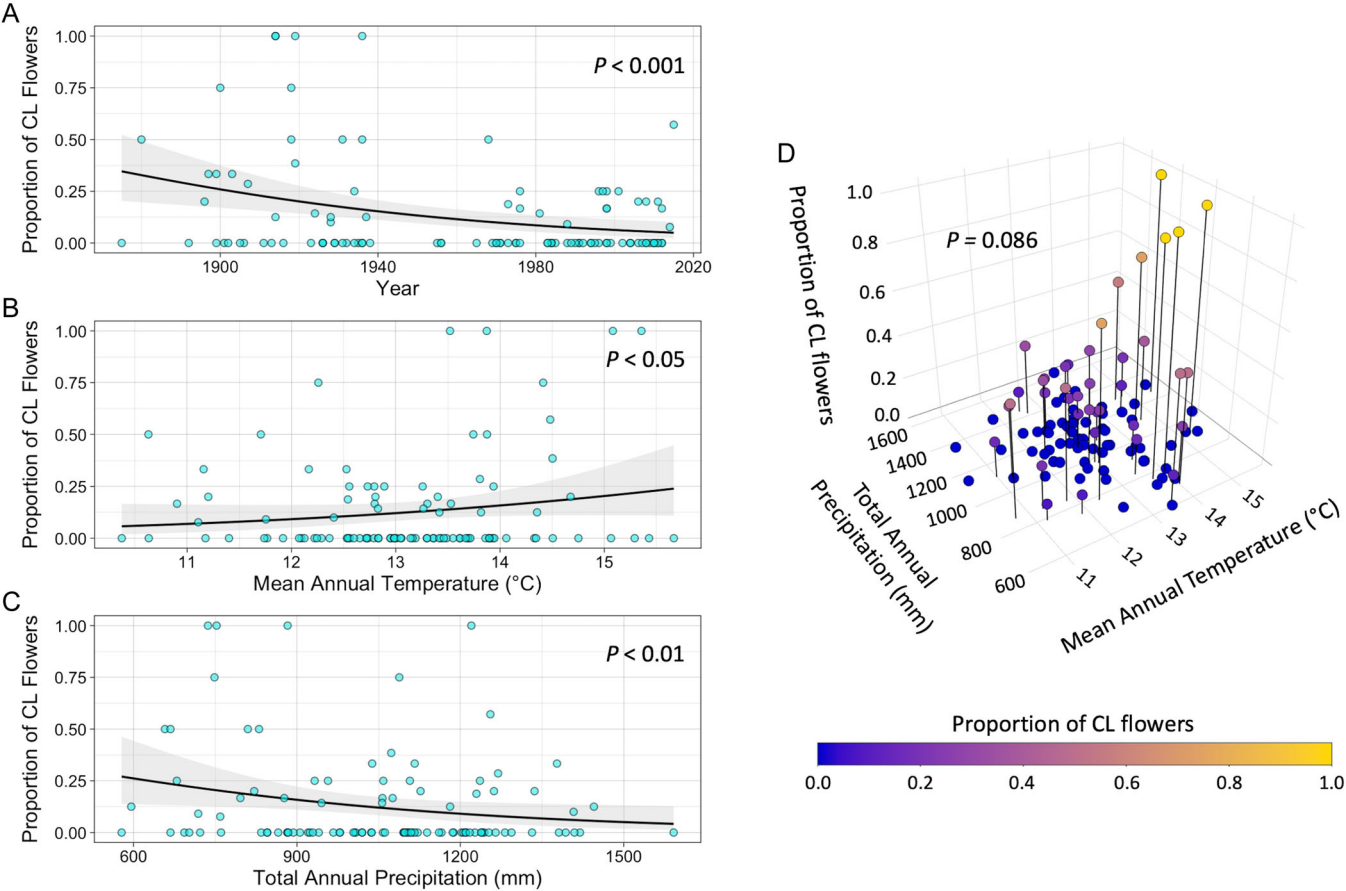
Association between climate change and reproductive strategy allocation of *Viola sororia*. The proportion of cleistogamous (CL) flowers (A) decreases from 1875 to 2015, (B) increases with higher mean annual temperature, and (C) decreases with higher total annual precipitation. Gray areas are 95% confidence intervals. (D) A marginally significant interaction between mean annual temperature and total annual precipitation reveals that chasmogamous (CH) flowers occur across a range of temperature and precipitation, while CL flowers predominantly occur when mean annual temperature is high and total annual precipitation is low.

*Viola sororia* plants produced fewer CL flowers relative to total flower number as the past century progressed. Across the past century, the proportion of CL flowers has decreased through time (*P* < 0.001; Table 1; Figure 4A). Moreover, *V. sororia* plants were more likely to produce lower proportions of CL flowers in years when temperature was low or precipitation was high. This effect is revealed by a significant positive correlation between the proportion of CL flowers and mean annual temperature (*P* < 0.05; Table 1; Figure 4B) and a significant negative correlation between the proportion of CL flowers and total annual precipitation (*P* < 0.01; Table 1; Figure 4C). A marginally significant effect was found for the interaction between mean annual temperature and total annual precipitation on the proportion of CL flowers (*P* = 0.086; Table 1; Figure 4D); high proportions of CL flowers tended to be more likely to occur in years with high temperature and low precipitation. These results are consistent with *V. sororia* having shifted away from production of obligately selfing CL flowers and toward the production of potentially outcrossing CH flowers as temperature has decreased and precipitation has increased across Missouri.

**Table 1:** Generalized linear models (GLM) results on the association between climate change and the proportion of cleistogamous (CL) flowers.

GLM, effect	Estimated coefficient (SE)	*t*	*P*
**Temporal model (*N* = 110)**			
Year	–0.017 (0.005)	–3.455	<0.001
**Climate model (*N* = 107)**			
Mean annual temperature	0.408 (0.191)	2.139	<0.05
Total annual precipitation	–0.003 (0.001)	–2.753	<0.01
Mean annual temperature × Total annual precipitation	0.002 (0.001)	1.734	0.086

Each model included the proportion of CL flowers as the response variable. Independent variables in the climate model were centered and scaled prior to analysis. For interpretability, the estimated coefficients and SEs for the climate model are reported back‐transformed, so that the estimated coefficients give the change in the proportion of CL flowers per unit change in the climate variables. SE = standard error.

### Is climate change associated with a shift in the time of year that CL and CH flowers occur?

**Figure 5:**
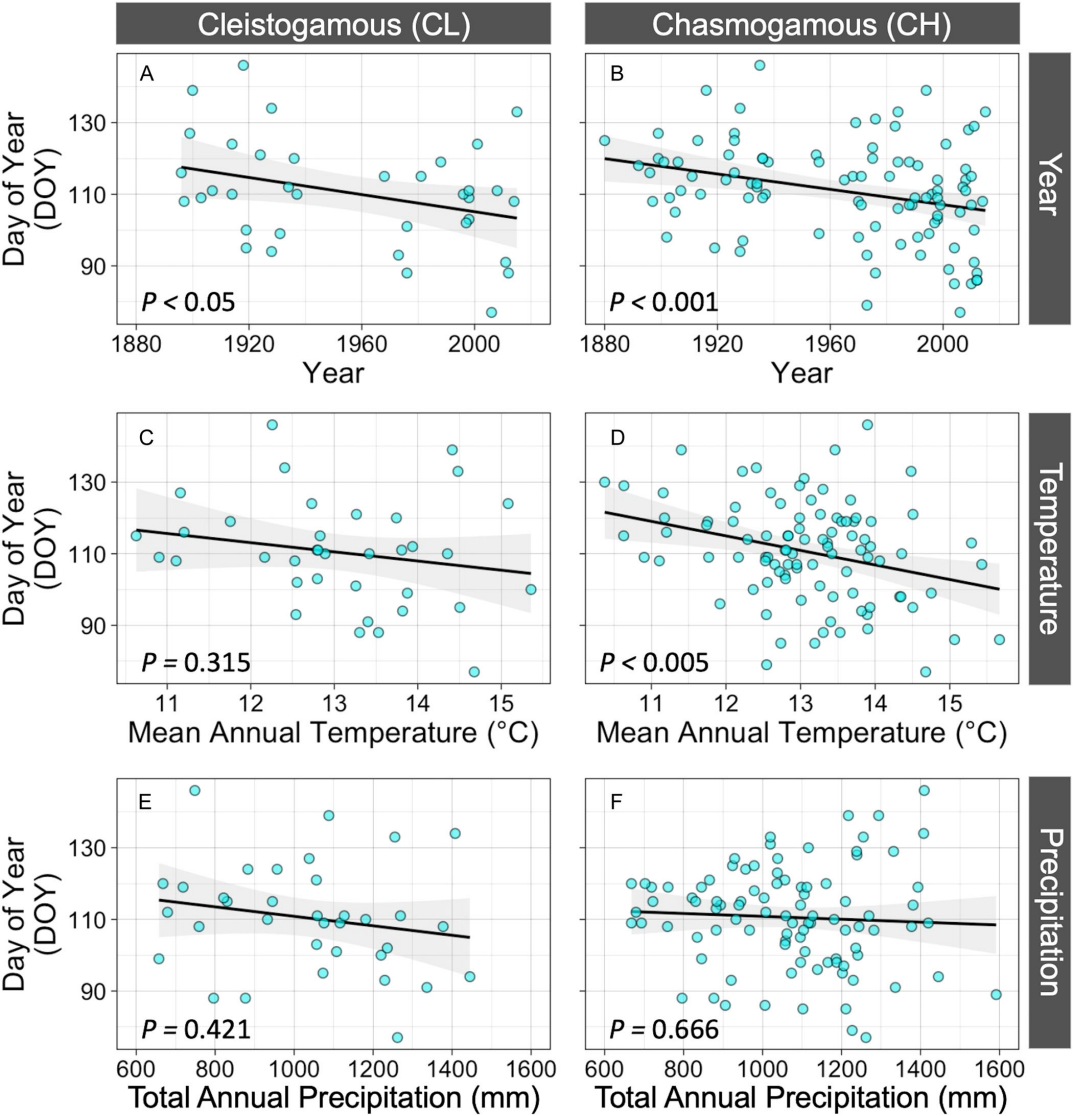
Association between climate change and the timing of cleistogamous (CL) and chasmogamous (CH) flowers. Flowering day of year (DOY) advances across the 20th century for both (A) CL and (B) CH flowers. Mean annual temperature (in degrees Celsius, °C) does not significantly affect CL flower DOY, but (D) does significantly affect CH flower DOY. Total annual precipitation does not significantly affect DOY for either (E) CL flowers or (F) CH flowers. Gray areas are 95% confidence intervals.

Both CL and CH flowers advanced their phenology across the past century (CL *P* < 0.05; CH *P* < 0.001; Table 2; Figure 5A, B). Spring‐occurring CL and CH flowers advanced by ~1.19 days (±0.58 SE) decade^−1^ and ~1.16 days (±0.34 SE) decade^−1^, respectively (Table 2).

**Table 2:** Generalized linear models (GLM) results on the association between climate change and the timing of cleistogamous (CL) and chasmogamous (CH) flowers.

GLM, effect	Estimated coefficient (SE)	*t*	*P*
**CL flowers**			
**Temporal model (*N* = 36)**			
Year	–0.119 (0.058)	–2.077	<0.05
**Climate model (*N* = 36)**			
Mean annual temperature	–2.277 (2.231)	–1.021	0.315
Total annual precipitation	–0.009 (0.011)	–0.816	0.421
Mean annual temperature × Total annual precipitation	–0.003 (0.011)	–0.241	0.811
**CH flowers**			
**Temporal model (*N* = 99)**			
Year	–0.116 (0.034)	–3.445	<0.001
**Climate model (*N* = 96)**			
Mean annual temperature	–4.060 (1.304)	–3.114	<0.005
Total annual precipitation	–0.003 (0.007)	–0.432	0.666
Mean annual temperature × Total annual precipitation	–0.005 (0.007)	–0.738	0.462

Each model included day of year (DOY) as the response variable. Independent variables in the climate models were centered and scaled prior to analysis. For interpretability, the estimated coefficients and SEs for the climate models are reported back‐transformed, so that the estimated coefficients give the change in DOY per unit change in the climate variables. SE = standard error.

We found that CH flowers bloom earlier in years where annual temperature is higher (*P* < 0.005; Table 2; Figure 5D), but timing of CH flowers was unrelated to total annual precipitation (*P* = 0.666; Table 2; Figure 5F) and the interaction between temperature and precipitation (*P* = 0.462; Table 2). Timing of CL flowering was unrelated to temperature, precipitation, and the interaction of these variables (all *P* > 0.31; Table 2; Figure 5C, E). These results suggest that the timing of obligately selfing CL flowers and potentially outcrossing CH flowers has shifted earlier across the past century, with phenological advancement of CH flowers being associated with temperature change.

## DISCUSSION

Integrating flowering and climate records across a 141‐year period, we find that the common blue violet (*Viola sororia*) has increased relative allocation to potentially outcrossing flowers as the climate has changed in Missouri over the past century. Specifically, we find that obligately selfing CL flowers progressively constituted a lower proportion of flowers on herbarium records from 1875 to 2015 and in environments with lower mean annual temperature and higher total annual precipitation (Figure 4A–C). Given that precipitation has increased, while temperature has vacillated yet decreased on average throughout Missouri across the 20th century (Figure 2A, B; EPA, 2016), these results suggest that *V. sororia* plants have responded to greater water availability and lower temperatures by increasing relative allocation to potentially outcrossing CH flowers. A marginally significant interaction between temperature and precipitation suggests that CL flowers occur in environments with high temperature and low water availability, while CH flowers occupy this entire climatic niche space (Figure 4D). We also find that *V. sororia* plants have advanced the timing of both CL and CH flowers across this time period (Figure 5A, B). To our knowledge, these results provide the first long‐term study of how anthropogenic climate change associates with reproductive strategy allocation, and as a result, opens a new window of inquiry into how global change may affect populations.

Effects of climate change on angiosperm reproduction are being documented by a burgeoning literature. Phenological alterations are perhaps the most extensively documented effect of climate change on floral reproduction, with warming temperatures generally driving earlier flowering (Visser and Both, 2005; Byers, 2017; Jones and Daehler, 2018). Our study builds upon these well‐documented changes to *when* reproduction occurs (Visser and Both, 2005; Byers, 2017; Jones and Daehler, 2018) by demonstrating that climate change can also potentially affect *how* reproduction occurs. Prior studies have suggested that species with mixed mating systems may be sensitive to anthropogenic global change (e.g., Jones et al., 2013) which, if true, is of particular consequence given that mixed mating occurs in approximately 42% of floral species worldwide (Goodwillie et al., 2005). For example, studies using manipulative climate perturbations (Jones et al., 2013) and comparisons between disturbed and undisturbed habitats (Eckert et al., 2010) suggest that when anthropogenic change induces stressful conditions, species with mixed mating systems respond by increasing relative allocation to selfing.

Our results suggest that climate change has altered the environmental conditions that *V. sororia* experiences in Missouri, which may have driven *V. sororia* to increase relative allocation to potential outcrossing. This is notable because outcrossing can increase effective recombination and potentially facilitate rapid adaptation in future generations (Stebbins, 1957). Climate change is manifesting nonuniformly across the globe, with changes to local climate often being different from the global average (IPCC, 2021). For example, the eastern two‐thirds of the contiguous United States experienced higher annual precipitation over the past 30 years relative to prior 30‐year normals, while the western one‐third experienced lower precipitation totals (NOAA, 2021). Responses of mixed mating systems are thus likely not uniform either. We predict that divergent responses of mixed mating systems have occurred between environments that have been disparately affected.

The climatic associations of obligately selfing CL flower production we observed are consistent with the interpretation that CL flowers are an adaptive strategy in environments with low resource availability, while potentially outcrossing CH flowers are produced under favorable growing conditions (Berg and Redbo‐Torstensson, 1998). Specifically, the positive effect of temperature and negative effect of precipitation on the proportion of CL flowers reveals that CH flowers are produced across a range of temperature and precipitation, while spring‐occurring CL flowers are more likely to occur when temperature is high and precipitation is low (Figure 4B, C). Interestingly, this creates open niche space where temperature is low and precipitation is high, in which CL flowers do not occupy (Figure 4D). These results implicate water availability as a key driver of investment in potential outcrossing in spring‐occurring *V. sororia*. In other words, the occurrence of obligately selfing CL flowers in environments with high temperature and low precipitation suggests a desiccating effect of high temperature in environments that are already water limited. This supports previous findings that CL flowers in other dimorphic cleistogamic species are more likely to be produced in environments with limited water and high temperature (Jones et al., 2013; Ansaldi et al., 2018).

Leveraging herbarium records as time series data, our study exemplifies the role biological collections can play in understanding long‐term effects of global change. Herbaria have been extensively used to study how climate change drives shifts in phenology (Jones and Daehler, 2018) and geographic distribution (Graham et al., 2004), and more recently have been leveraged to study effects on herbivory (Meineke et al., 2019), pollination (Johnson et al., 2019), and floral pigmentation (Koski et al., 2020), among other topics. In this study, we demonstrate that for mixed mating species with dimorphic flowers, herbaria can also be used to investigate changes in reproductive strategy allocation. While this is a powerful approach, it is not without its limitations. For example, herbarium records are neither systemically sampled nor are they a completely randomized collection of plants; botanists tend to collect plants early in their reproductive season (Daru et al., 2018) (albeit oftentimes after populations have begun flowering; Pearse et al., 2017), with a bias toward uncommon plants (Garcillán and Ezcurra, 2011). Furthermore, to aid taxonomic classification, botanists likely often prefer to sample plants containing reproductive structures (Daru et al., 2018). The observed decrease in the proportion of CL flowers across the 20th century could result from modern collectors being more biased toward collecting CH flowers than were collectors in the historic past. Additionally, our herbarium data cannot directly address whether a bias toward potential outcrossing in cooler and wetter conditions occurs because of increased production of CH flowers, decreased production of CL flowers, or if both occur while the total flower number remains constant. However, prior research in the dimorphic cleistogamic species *Oxalis acetosella* has found that reproductive output is equivalent between individuals that differ in their relative allocation to potential outcrossing vs. selfing; i.e., total seed number per ramet does not differ between individuals that produce high and low proportions of CL flowers (Berg and Redbo‐Torstensson, 1998). In addition, the use of herbarium specimens in our study prohibits us from knowing whether the progeny of the observed CH flowers would have been produced via outcrossing or autogamy. Future research should explore the mechanistic basis of how temperature and water availability drive reproductive strategy allocation in *V. sororia*, while accounting for changes in total reproductive output and CH flower autogamy.

We restricted our analyses to the spring period of *V. sororia*'s reproductive season for two reasons. First, this captures the period of overlapping CH and CL flower production as plants transition from predominantly CH flowering to predominantly CL flowering (Figure 3B). Second, herbarium records of *V. sororia* are biased to this spring period; only sparse sampling of *V. sororia* was undertaken in the summer and fall (Figure 3A). Accordingly, our results only reveal the potential effect of climate change on *V. sororia*'s flowering in a portion of their annual flowering season. Notably, when we use data across *V. sororia*'s entire flowering season to test for an association between climate change and the proportion of CL flowers (i.e., rerunning our models that test for a shift in reproductive strategy allocation using herbarium records from the spring and summer‐fall), we still find a decrease in the proportion of CL flowers across the past century (*P* < 0.01), but no longer find a significant association between the proportion of CL flowers and mean annual temperature (*P* = 0.219), total annual precipitation (*P* = 0.080), or their interaction (*P* = 0.227). While sparse sampling in the summer‐fall limits our ability to interpret trends in this latter period of *V. sororia*'s flowering season, these results are consistent with (1) CL flowering having decreased relative to CH flowering across the entire growing season (as opposed to the decrease in spring CL flowering being compensated for by increased CL flowering in the summer‐fall), and (2) flowering in the summer‐fall being governed by environmental factors different from those governing flowering in the spring (e.g., similar to other *Viola* species, flowering in the summer and fall may be governed by light levels, as opposed to climate; Culley, 2002).

Importantly, our results demonstrate that the observed temporal changes to the proportion of CL flowers are not due to one flower type having altered their phenology, while the other has not. Rather, we find that timing of both spring‐occurring CL and CH flowers has advanced across the past century (Figure 5A, B). These findings add to a growing body of literature on how climate change drives phenological advancement (e.g., Menzel et al., 2006; Miller‐Rushing and Primack, 2008; Calinger et al., 2013; Davis et al., 2015; Jones and Daehler, 2018) and, to the best of our knowledge, provides the first documentation of obligately selfing CL flowers having advanced across the 20th century. Interestingly, while we find that both flower types have progressively occurred earlier across this time period, climate was only associated with phenology shifts of CH flowers; CL phenology shifts were not associated with either mean annual temperature or total annual precipitation. Additionally, it is notable that CH flower advancement is associated with warming temperatures, i.e., CH flowers occur earlier in years with warmer mean annual temperature (Figure 5D), despite Missouri having experienced a decrease in mean annual temperature on average across the past century (Figure 2A). It is likely that changes to Missouri's climate not captured by mean annual temperature and total annual precipitation have contributed to the advancement of CL and CH flowers in *V. sororia*. Future research is needed to clarify how phenology of mixed mating systems is governed by climate, particularly in systems subject to rapid environmental changes. Moreover, given that outcrossing of CH flowers is pollinator‐mediated, future research should explore whether CH flower advancement in *V. sororia* has driven phenological mismatch with pollinators, and if so, how such mismatch affects realized outcrossing rates.

## CONCLUSIONS

Our results suggest that anthropogenic global change is associated with reproductive strategy allocation in species with mixed mating systems. Using climate change as a temporal gradient, our results support selfing as an adaptive strategy that provides reproductive assurance in resource‐limited environments, while outcrossing is employed in resource‐rich environments (Becerra and Lloyd, 1992; Goodwillie et al., 2005). Furthermore, by using herbarium records as time series data across the 20th century, this study demonstrates the utility of using biological collections for addressing the effects of global change, particularly when long‐term field collected data are unavailable. Future research should explore if mixed mating systems experiencing disparate effects of climate change are divergently altering relative allocation to potential outcrossing vs. selfing. Additionally, as reproductive strategy allocation affects genetic diversity and the ability of populations to adapt to future change, research should explore the population genetic consequences of altered reproductive strategy allocation. It would be informative to expand this research to other mixed mating species, including plants with hermaphroditic flowers where selfing syndrome traits (e.g., reduced herkogamy, lower pollen‐to‐ovule ratios) can be used to elucidate reproductive strategy allocation (Sicard and Lenhard, 2011). In an era where human activity exerts a dominant influence on the global environment, ensuing changes to the climate are an added temporal dimension to the factors influencing reproductive strategy allocation.

## AUTHOR CONTRIBUTIONS

M.W.A. conceived the idea and led the writing of the manuscript; M.W.A., K.M.O., and A.B.S. designed the methodology; M.W.A. and P.O.C. collected and analyzed the data. All authors contributed critically to the drafts and gave final approval for publication.

## CONFLICT OF INTEREST

The authors declare no conflicts of interest.

## Supporting information


**Appendix S1.** Map of *Viola sororia*'s range and study area; seasonality of Missouri's climate; frequency of *V. sororia* herbarium records across the 20th century.

## Data Availability

Data underlying analyses can be found on the Dryad Digital Repository: https://doi.org/10.5061/dryad.tb2rbp032 (Austin et al., 2022).
