# Supplementary material for: Climate change is associated with increased allocation to potential outcrossing in a common mixed mating species
Source: Am J Bot. 2022 Jun 28;109(7):1085–96. doi: 10.1002/ajb2.16021 (PMC9544429; doi:10.1002/ajb2.16021)
Supplement: Supplementary file 1 — Appendix S1. Map of Viola sororia's range and study area; seasonality of Missouri's climate; frequency of V. sororia herbarium records across the 20th century. [file AJB2-109-1085-s001.pdf]

American Journal of Botany

Climate change is associated with increased allocation to potential outcrossing in a common mixed mating species

Matthew W. Austin[1,*], Piper O. Cole[2], Kenneth M. Olsen[3], Adam B. Smith[4]

[1] Living Earth Collaborative, Washington University in St. Louis, St. Louis, MO, USA
[2] Division of Natural Sciences, New College of Florida, Sarasota, FL, USA
[3] Department of Biology, Washington University in St. Louis, St. Louis, MO, USA
[4] Center for Conservation and Sustainable Development, Missouri Botanical Garden, St. Louis, MO, USA

*** Corresponding author:**
Washington University in St. Louis
320 McDonnell Hall, Danforth Campus
St. Louis, MO, USA 63105
AustinMattW@gmail.com
ORCID ID: 0000-0002-1231-9081

Table of Contents

Fig S1. Map of *Viola sororia*'s range and study area ____ 2
Fig S2. Seasonality of Missouri's climate ____ 3
Fig S3. Frequency of *V. sororia* herbarium records across the 20th century ____ 4
References for Appendix S1 ____ 5

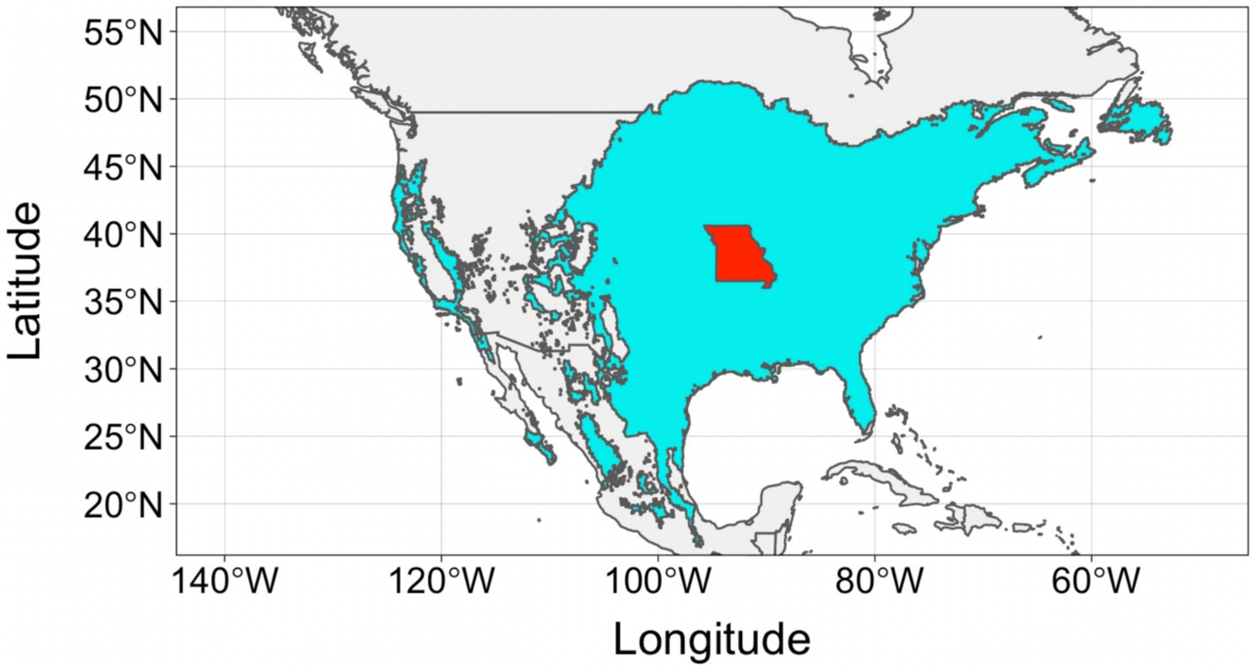

Figure S1. Range map of *Viola sororia* (blue), with Missouri highlighted (red). Range map obtained from the *BIEN* package (Maitner et al. 2018).

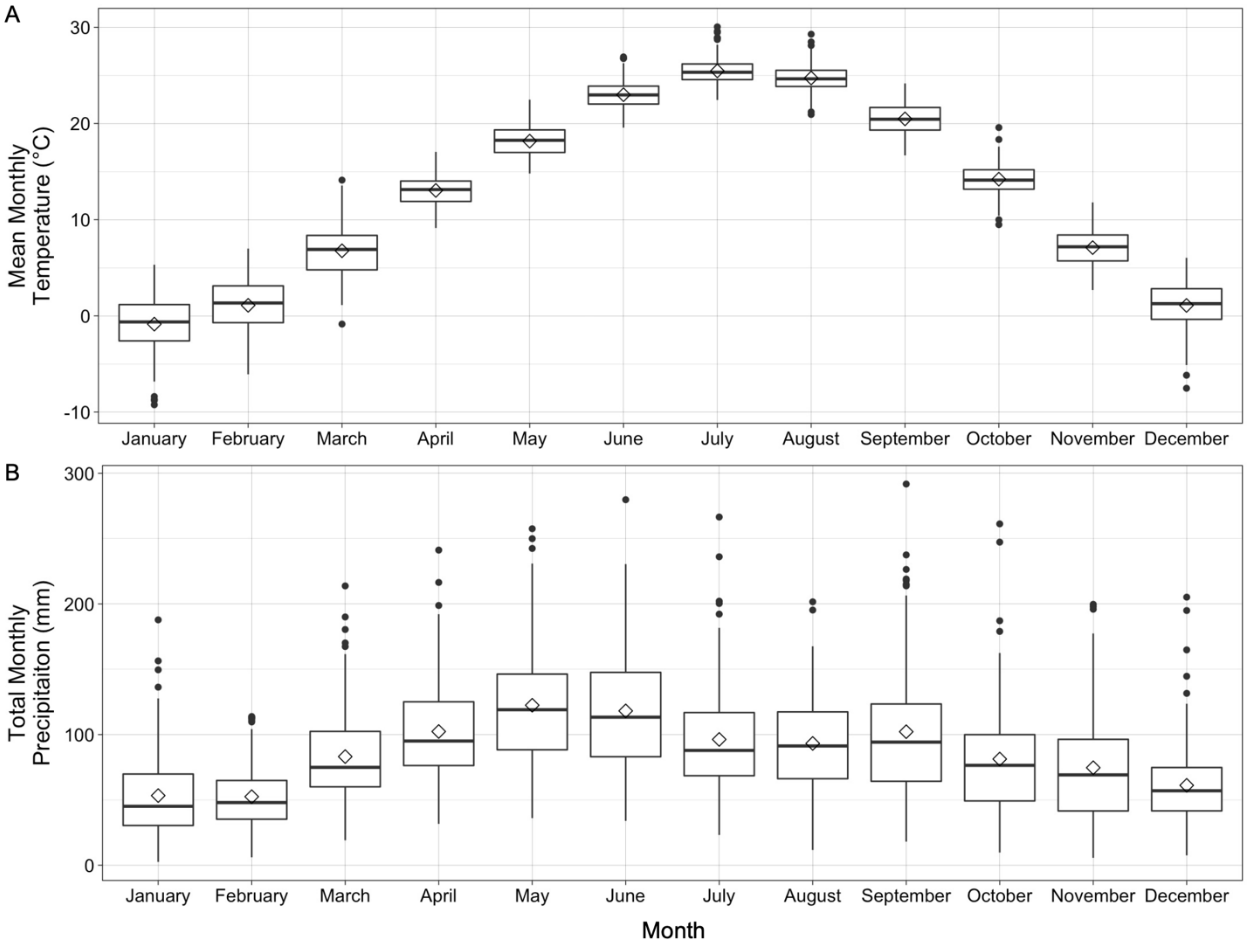

Figure S2. Seasonality of Missouri's temperate climate. Box plots show monthly ranges of state-wide averages of (A) mean monthly temperature (°C) and (B) total monthly precipitation (mm) from 1895 to 2015 for 1,000 coordinates randomly sampled across Missouri. Horizontal bars are medians; diamonds are means. Climate data derive from the Parameter-elevation Regressions on Independent Slopes Model (PRISM) (Daly et al. 2002).

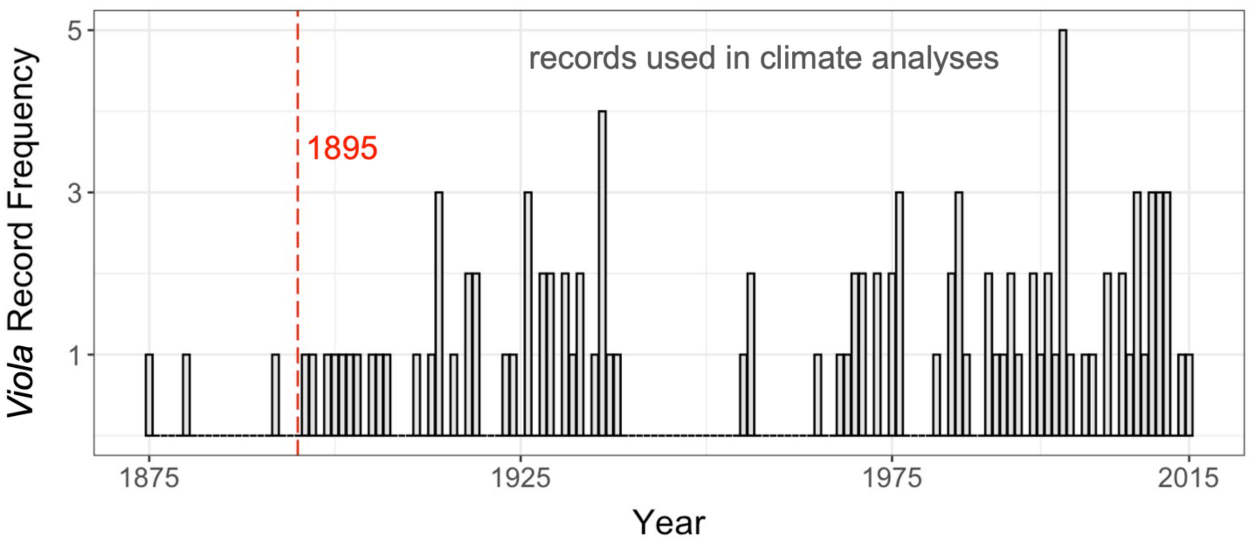

Figure S3. The frequency of herbarium records across the 20th century; the dashed red line indicates 1895, which is the first year climate data were available for.

References for Appendix S1

Daly, C., Gibson, W.P., Taylor, G.H., Johnson, G.L., & Pasteris, P. 2002. A knowledge-based approach to the statistical mapping of climate. Climate Research 22: 99-113.

Maitner, B.S., Boyle, B., Casler, N., Condit, R., Donoghue, J., Durán, S.M., Guaderrama, D., Hinchliff, C.E., Jørgensen, P.M., Kraft, N.J., & McGill, B. 2018. The bien r package: A tool to access the Botanical Information and Ecology Network (BIEN) database. Methods in Ecology and Evolution 9: 373-379.
